# Nano toolbox in immune modulation and nanovaccines

**DOI:** 10.1016/j.tibtech.2022.03.011

**Published:** 2022-10

**Authors:** Mohammad Azharuddin, Geyunjian Harry Zhu, Anirban Sengupta, Jorma Hinkula, Nigel K.H. Slater, Hirak K. Patra

**Affiliations:** 1 Department of Biomedical and Clinical Sciences (BKV), Linköping University, Linköping, Sweden; 2 Department of Chemical Engineering and Biotechnology, University of Cambridge, Cambridge, UK; 3 Department of Surgical Biotechnology, University College London, London, UK

**Keywords:** vaccine, nano toolbox, nanovaccine, immune modulation, immune response

## Abstract

Despite the great success of vaccines over two centuries, the conventional strategy is based on attenuated/altered microorganisms. However, this is not effective for all microbes and often fails to elicit a protective immune response, and sometimes poses unexpected safety risks. The expanding nano toolbox may overcome some of the roadblocks in vaccine development given the plethora of unique nanoparticle (NP)-based platforms that can successfully induce specific immune responses leading to exciting and novel solutions. Nanovaccines necessitate a thorough understanding of the immunostimulatory effect of these nanotools. We present a comprehensive description of strategies in which nanotools have been used to elicit an immune response and provide a perspective on how nanotechnology can lead to future personalized nanovaccines.

## Highlights

Nanoscale materials can extend antigen stability, enhance immunogenicity, and improve antigen presentation time in the targeted cell or tissue.
The reasons behind the current success of advanced nanoscale vaccine technologies and how they differ from traditional and conventional vaccines in terms of immune modulation are discussed.
The capacity and extent of eliciting humoral and cell-mediated immune responses by nanovaccines are reviewed
We present a list of all currently FDA-approved nanovaccines and those in clinical trials.
An in-depth and rational understanding will be necessary for the development of nanotools for use in future vaccines. We overview the lessons learnt from this potentially transformative nanovaccine development and how they have been used to elicit an immune response, with a focus on the most recent nanovaccines.

## Nanoscale improvements to traditional vaccines

The immune system is an interconnected mesh of cells, tissues, and organs that protect the body against fatal diseases. Immune homeostasis is disrupted by either an underperforming or hyperactive immune response; the former can fail to protect against a simple infection [1] whereas the latter can result in destruction of healthy tissue [2,3]. The immune system consists of innate (non-specific) and adaptive (specific) immunity. Adaptive immunity is characterized by its ability to precisely identify a pathogenic substance and to develop a long-term memory of it. Vaccines train the adaptive immune system to either generate immunological memory before infection (prophylactic) or to recognize ongoing disease (therapeutic) [4]. Although the development of prophylactic vaccines against fatal infections such as smallpox, anthrax, and plague has made a very significant contribution to healthcare, more recent advances in therapeutic vaccines provide promise for treating incurable conditions such as cancer, HIV infection, and type I diabetes [5]. Conventional vaccines based on attenuated or inactivated pathogens suffer from the potential risk of introducing live pathogens and the inability to elicit a satisfactory level of immunity, thus stimulating the development of new vaccines [6]. With progress in nanotechnology, NP-based vaccines (nanovaccines) have been formulated that not only overcome the drawbacks of traditional vaccines but also afford advanced-level modulation that was not previously possible [7., 8., 9.]. Superior efficacy can be achieved by nanovaccines because of (i) extended antigen stability, (ii) enhanced immunogenicity, (iii) targeted delivery, and (iv) sustained release (Box 1).

Box 1 **Key features of nanovaccines**

**Extended antigen stability:** because of the protective nature of the NPs, the antigens are protected from degradation by cellular components and enzymes.

**Enhanced immunogenicity:** the NPs themselves can be immunogenic, leading to an enhanced immune response against the target antigen.

**Targeted delivery:** nanovaccines can be designed to deliver antigen to targeted sites such as specific cell types or tissues, and thus reduce the likelihood of harmful side effects.

**Protection of antigens and adjuvants against enzymatic and proteolytic degradation:** key immunogenic components such as peptides, oligonucleotides, and adjuvants are protected from degradation by the nanovaccine formulation.

**Evoke both humoral and cell-mediated immune responses:** the two major branches of immunity (the antibody and cellular responses) can both be enhanced by nanovaccines.

**Present multiple components in a single platform:** multiple antigens can be included in the same NP, leading to a nanovaccine formulation that can potentially protect against a wider range of antigens or infections.

**Enhanced duration of antigen presentation and DC processing:** professional APCs require time to recognize and process antigen before presenting it to elicit a downstream immune response. Nanovaccines can persist for a longer time without alteration or degradation and thereby provide ample opportunity for APCs to boost the immune response.

Alt-text: Box 1

**Figure 1:**
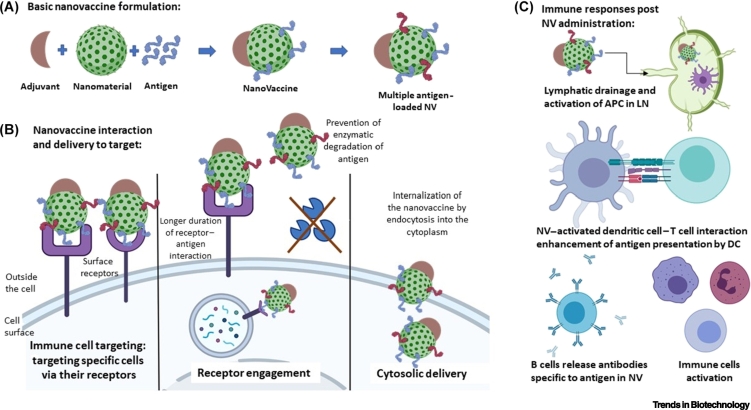
The basics of nanovaccines and their significance. (A) Nanovaccines comprise a selected antigen conjugated to a nanomaterial and an adjuvant to elicit immunogenic response. Multiple antigen epitopes (denoted by red and blue antigens) can be loaded onto the surface of the NPs. Nanomaterial and adjuvant types vary depending on the infection, tissue type, and the immune response required. (B) NPs aid efficient vaccine targeting to the desired cell and its receptors, thereby minimizing side effects. They increase the duration of antigen-receptor engagement and thus enhance the immune response. Specific types of NPs are useful in delivering the antigen into the cytoplasm of the target cell. Packaging of antigens within NPs enhances their protection against enzymatic or proteolytic cleavage. (C) NPs can pass through the lymphatic drainage system and activate APCs within the lymph nodes. (D) NPs aid the DC–T cell interaction that is necessary to boost the downstream immune response. They activate dendritic cells and influence the release of pro- and anti-inflammatory cytokines. (E) Antibody production by plasma B cells and the differentiation, maturation, and activation of lymphocytes and monocytes is also positively influenced by NP-mediated vaccine delivery. Abbreviations: APC, antigen-presenting cell; DC, dendritic cell; LN, lymph node; NP, nanoparticle; NV, nanovaccine.

NPs can provide strong protection to both the antigens and adjuvants against enzymatic and proteolytic degradation [10]. NPs can evoke both humoral and cell-mediated immune responses because of their unique physicochemical characteristics (Figure 1). They also aid in targeted delivery and can potentially load multiple antigenic components into a single platform [11., 12., 13., 14., 15., 16.]. Lastly, fine-tuning the physical attributes such as size, shape, and surface charge of the NPs can lead to substantial enhancement in the duration of antigen presentation and dendritic cell (DC)-mediated antigen uptake, leading to mature DCs and promoting cell-mediated immunity [17., 18., 19.]. We review how different nanotools have been utilized successfully for improving immunogenicity and developing novel vaccines. The specific role of NPs in vaccine improvement with respect to their size, loading efficiency, nano-enhanced immunogenicity, antigen presentation, and retention in lymph nodes (LNs) is discussed. Finally, nanovaccines that are approved for clinical use or under clinical investigations are summarized.

## Types of nanomaterials

NPs are ideal vehicles to deliver antigens for vaccination because they are comparable in size to viruses and have the ability to load and release active biomolecules [20]. Many types of NPs have been utilized to develop nanovaccines, including metallic NPs, carbon nanotubes, liposomes, micelles, dendrimers, and biomacromolecules. Noble metal NPs, such as colloidal gold, are bio-inert, nontoxic, and their synthesis is well established [21]. Gold NPs (AuNPs) have been utilized for vaccines against influenza [22], malaria [23], and cancer [24]. However, their long-term accumulation remains a safety concern [25]. Other inorganic NPs which have been utilized in vaccine formulations include carbon nanotubes [26], silica NPs [27], and magnetic NPs [28]. Polymeric materials have been widely explored as nanovaccines because of their desirable biodegradability and biocompatibility. Polylactide-co-glycolic acid (PLGA) copolymer [29,30], chitosan [31], and other types of in-house synthesized polymers [32., 33., 34.] have been shown to successfully deliver antigens. Micelles [35., 36., 37.], liposomes [38,39], and dendrimers [40,41] have been investigated as nanovaccines based on their ability to load and deliver antigens. Although proteins usually serve as the antigens in subunit vaccines, engineered proteins can self-assemble into antigen-containing NPs and act as nanovaccines [42,43].

Nanovaccines exploit NP drug delivery systems in general, and biocompatibility and safety are major metrics. Although the goal of nanovaccines is to elicit a specific immune response, it is important that their immunogenicity is antigen-specific rather than NP-specific [44]. By contrast, adjuvanticity – the ability to augment the immune response – is desirable for NPs in nanovaccine formulations. It has been demonstrated that NPs made from a wide range of materials can promote an immune response, including those composed of materials that are widely considered to be biocompatible [45]. There is growing evidence that metallic NPs (e.g., gold, iron, and nickel) display immune-modulatory properties by promoting cell recruitment, antigen-presenting cell (APC) activation, and cytokine induction, and can facilitate a humoral response. Niikura and coworkers showed that spherical AuNPs of 40 nm in diameter, surface-modified with West Nile envelope protein (WNE), produced the highest titers of WNE-specific antibodies and also induced inflammatory cytokine production, including tumor necrosis factor-α (TNF-α), interleukin (IL)-6, IL-12, and granulocyte macrophage colony-stimulating factor (GM-CSF) [46]. Citrate-stabilized AuNPs ranging from 2 to 50 nm in diameter conjugated with a synthetic peptide for a foot and mouth disease virus (FMDV) protein showed higher antibody titers for NPs in the 8–17 nm size range, and other spherical AuNPs (<50 nm) have been reported as antigen carriers for immunization against several other microorganism [22,47., 48., 49., 50., 51., 52., 53., 54., 55., 56.].

## Size-dependent immunogenicity

Antigens delivered by NPs are known to elicit stronger antigenic responses compared to their free counterparts because of the combination of enhanced stability, sustained release, and adjuvant effects [57., 58., 59.]. NP size is a crucial factor that can strongly influence the efficacy and ultimately affects the magnitude and type of immune response (B cell vs. T cell) [60]. Particles with a size of >1 μm (i.e., comparable in size to a bacterial pathogen) are internalized via phagocytosis, whereas smaller particles <1 μm in size are internalized by micropinocytosis, receptor-mediated clathrin-coated endocytosis, and clathrin-independent and caveolin-independent endocytosis [61., 62., 63.]. Thus, particle size is a determining factor that dictates NP entry, the intracellular fate of the antigen processing, and T cell activation. It was recently revealed that small NPs have a higher uptake efficiency by DCs [18,60,64] and accumulate in the LNs with greater efficacy than large NPs, thus inducing an enhanced immune response [65]. However, a universal correlation between size and immune response for solid particle-based NPs has not been reached [66,67], and NPs composed of different core materials showed various optimum sizes for the induction of an immune response [68]. In general, smaller particles are considered to be more effective for targeted drug delivery because of their improved ability to permeate biological barriers [69,70]. Conversely, for a nanovaccine formulation, these criteria do not hold true because the purpose of vaccination is to elicit a designated immune response by allowing specific recognition by the immune system. To date, agreement on the optimum nanovaccine size range that generates a stronger immunological response has not been achieved [64].

For example, 1000 nm bovine serum albumin (BSA)-loaded PLGA particles evoked a more robust serum IgG response than particles sized 200–500 nm [66]. By contrast, some researchers report that smaller NPs are more efficient and potent immune system stimulators. For instance, an NP-based nicotine vaccine consisting of PLGA and a lipid shell produced a significantly higher anti-nicotine antibody (IgG1 and IgG2) titers with a 100 nm than a 500 nm nanovaccine [71]. One possible explanation is a difference in the mechanism of immunity that is targeted. Large-sized nanomaterials boost humoral immune responses, whereas smaller NPs promote cell-mediated immune protection [72., 73., 74.]. Larger NPs have a tendency to preferentially generate type 2 T helper (Th2) cell responses [7,75,76]. This is mostly because of differential uptake – for sizes >500 nm the internalization and processing of antigen leads to a more efficient presentation by MHC II, thereby generating a stronger humoral response [7,75]. For example, a study showed that smaller HIV TAT protein-modified cationic polymeric NPs promote a higher TAT-specific cellular immune response and a weaker anti-TAT antibody response than larger particles (~2 μm) [77]. In another study, using poly-lactic acid (PLA)-entrapped hepatitis B virus surface antigen (HBsAg), a single immunization with smaller particles induced a lower humoral response than did larger particles [74]. Immunization with smaller particles encouraged Th1 immune responses, whereas the larger particles favored Th2 responses [74]. This is because the smaller particles were efficiently engulfed by APCs such as macrophages, which leads to cellular immune response, whereas larger particles cannot be taken up by macrophages but can adhere to the macrophage surface and release trapped antigens.

Another study showed that nanobeads of 40–49 nm could evoke the secretion of Th1-biased cytokines, whereas nanobeads of 93–101 nm elicited Th2-biased cytokine secretion following immunization in mice [78]. These observations showed that precise selection of NP size for vaccination can influence the type1/type2 cytokine balance, which can be crucial for protection against respiratory syncytial virus [78]. Similarly, polystyrene beads of 40–50 nm effectively induced cellular responses by activating CD8^+^ T cells and interferon (IFN)-γ production [79]. This was tested in an *in vivo* animal model where polystyrene beads of 48 nm covalently bound to antigen induced an enhanced antigen-specific Th1-biased response and IFN-γ production [80]. Other studies show that NPs of larger size can also induce a robust Th1 response with predominant IFN-γ production by priming CD4^+^ T cells [81,82]. Researchers have shown that large bile salt-stabilized vesicles (bilosomes) with influenza A antigens elicited immune responses that were biased toward Th1 as compared to small particles [83]. Given such variability, it is difficult to predict the optimum NP size range to elicit a Th1 or a mixed Th1/Th2 immune response. Finally, the kinetics of NP migration through the lymphatic vessels is highly size-dependent [65,84,85]. Particles <5 nm in size can freely enter the bloodstream whereas particles of >100 nm remain at the injection site and fail to move into the lymphatic system. LN targeting is discussed in detail in a later section. Table 1 summarizes the size-dependency of nanosystem immunological responses.

**Table 1:** Effect of NP size on the immunological response

Size	Material	Context	Immunological response	Refs
1.5 nm	Gold	*Listeria*	AuNP–LLO (listeriolysin O peptide) plus Advax™ adjuvant induced LLO-specific T cell immunity and protection against *Listeria* challenge	[47]
2–50 nm	Gold	Foot and mouth disease	Specific antibodies were induced by 2, 5, 8, 12, and 17 nm FMDV plus cysteine (pFMDV)–AuNP conjugates. Maximal antibody titer was generated with 8–17 nm conjugates	[48]
10–100, 60–350, 400–2500 nm	Bilosome	Influenza	Larger bilosome particles with influenza A antigens elicited immune responses that had a significantly greater Th1 bias than the small particles	[83]
12 nm	Gold	Influenza	Matrix 2 protein (M2e)–AuNP conjugates induced M2e-specific IgG serum antibodies	[22]
20–123 nm	Polystyrene	Respiratory syncytial virus (RSV)	IFN-γ induction from CD8 T cells was limited to 40−49 nm beads, whereas CD4 T cell activation and IL-4 were induced by 93−123 nm beads	[78]
30–200 nm	Polystyrene	Tumor	Nanobeads of 40–50 nm effectively induced cellular responses by activating CD8^+^ T cells with IFN-γ production	[79]
40 nm	Gold	Tetanus toxoid	Enhanced tetanus toxoid (TT)-specific IgG (34.53×) and IgA (43.75×) was elicited by TT-ARE-CsAuNPs	[49]
100, 500 nm	PLGA	Nicotine	The 100 nm particles induced significantly higher antibodies than the 500 nm particles	[71]
200, 500, 1000 nm	PLGA	Bovine serum albumin	A greater IgG response was elicited by 1000 nm particle than by 200–500 nm particles	[66]
200–600 nm, 2–8 μm	PLA	Hepatitis B virus	Hepatitis B virus surface antigen (HBsAg) encapsulated in 2–8 μm particles generated more antibodies than 200–600 nm particles	[74]
220, 660, 1990 nm	PMMA Eudragit®	HIV	HIV TAT protein modified NPs of 220 or 630 nm elicit strong TAT-specific cellular immune response but weaker anti-TAT antibody response than NPs of 1.99 μm	[77]

## NP loading of antigens

Antigens of interest can be either encapsulated within or attached to the surface of NPs. Antigen encapsulation can be achieved with polymeric, micellar, and liposomal NPs [86], and surface functionalization can be performed with polymeric, inorganic, or metallic NPs [67,79,87,88]. In general, encapsulation of antigens into NP cores gives protection against enzymatic degradation, whereas surface immobilization mimics the presentation of antigens by pathogens [89]. More recent studies have focused on using biomimetic strategies to load antigens, such as by using lipid membranes. Liu and colleagues reported the fabrication of self-assembled nanovaccines containing phospholipids which were able to deliver strong initial antigen stimulation followed by controlled long-term antigen release, leading to effective cross-presentation and a CD8^+^ T cell response [90]. When choosing the loading method, multiple factors including loading capacity, release efficiency, preservation of antigen function and structure, epitope orientation, and the overall influence on the colloidal stability of the NPs [91] must be carefully considered.

**Figure 2:**
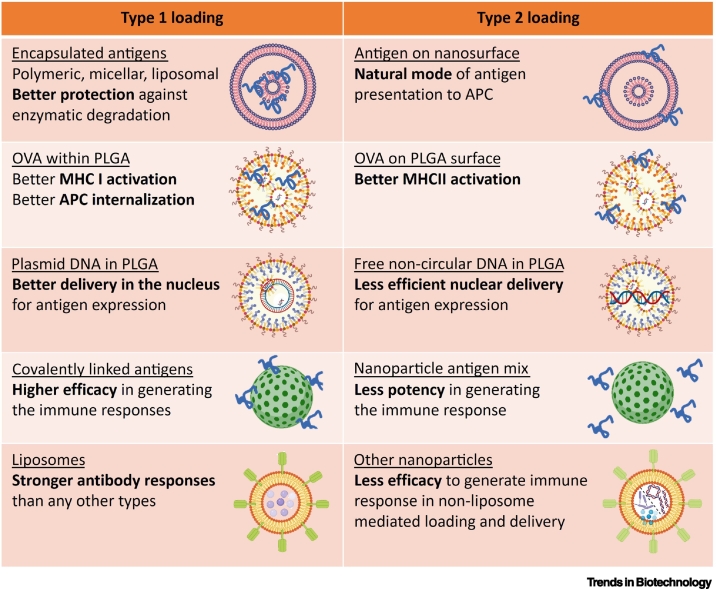
Comparative account of loading strategies of nanoparticles to boost the immune response. Different modes of loading of the nanomaterials to enhance immune responses. It was previously reported that encapsulation of antigens tends to provide better protection [89], better MHC I activation [92], and better nuclear delivery [100] than surface immobilization. Covalently attached antigens can generate stronger immune responses than non-covalently tethered antigens [79]. Liposomes can elicit stronger antibody responses than other nanoparticle systems [57,93., 94., 95., 96., 97., 98., 99.]. Abbreviations: APC, antigen-presenting cell; OVA, ovalbumin; PLGA, polylactide-co-glycolic acid.

To date, there are limited systematic studies on the effect of loading methods on nanovaccine efficiency. One reason is that the loading method is often specific to the NP system of choice, such as surface functional groups, geometry structure, and fabrication technique (Figure 2). It was found that chemically conjugated protein antigen induced a stronger immune response than when the same antigen was simply physically mixed with the NPs, but this was possibly because of different loading capacities [79]. A study comparing PLGA NPs with encapsulated versus surface-adsorbed ovalbumin (OVA) demonstrated that faster *in vitro* internalization was achieved by the encapsulation architecture; however, the difference might be caused by a change in surface charge [92]. In addition, it was revealed that PLGA NPs with encapsulated OVA preferentially activated the MHC I pathway as compared to PLGA NPs with surface-adsorbed OVA which resulted in enhanced MHC II presentation [92]. Several other reports imply that liposomes with covalently conjugated antigens generate stronger antibody responses than other types of loading strategies [57,93., 94., 95., 96., 97., 98., 99.]. For DNA vaccines, there have been reports that plasmid DNA vaccine adsorbed onto PLGA NPs was much more efficient than the same DNA entrapped inside PLGA [100]. In DNA vaccines, the nanocarriers serve as the non-viral vector for gene delivery (as reviewed extensively elsewhere [101,102]). To sum up, it seems the surface-loading method has some advantages over the entrapment method, but more systematic studies with various nanosystems should be conducted to provide a clearer picture.

## Nano-enhanced immunogenicity and antigen delivery

**Figure 3:**
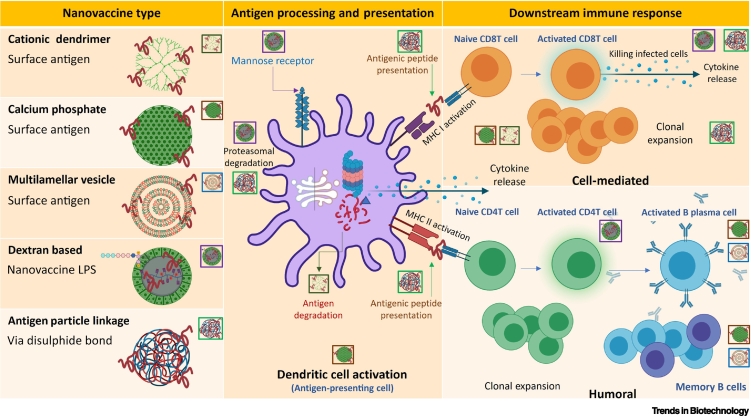
Mechanism of action of nanovaccines. Different types of antigens conjugated to nanoparticles (NPs) stimulate antigen-presenting cells (APCs) to process and present the antigens in different manners. Some antigens are received by mannose receptors, some are degraded within the APCs and the antigenic peptide fragments are then presented via MHC I (to activate CD8 T cells) or via MHCII (to activate CD4 T cells). APCs (like dendritic cells and T cells) also secrete cytokines in the process. This release of cytokines alters the cytokine milieu and shapes either pro- or anti-inflammatory responses. Clonal expansion of the activated T cells and B cells leads to boosting of the immune response. Activated plasma B cells release antibodies in response to the specific antigen conjugated to the NPs. Some cells remain as memory cells to provide an immediate antibody response in the case of natural antigenic challenge. The annotations adjacent to individual nanovaccines highlight mechanistic steps taking place in APCs or downstream immune response column and illustrate the diverse mechanisms of action of individual nanovaccines. Abbreviation: LPS, lipopolysaccharide.

Antigens delivered by NPs are internalized through several endocytic pathways. Apart from the size effect discussed above, surface charge and surface functionalization of targeting molecules can facilitate delivery to APCs for antigen presentation. Cationic NPs are internalized by APCs more rapidly and usually promote intracellular trafficking through endosomal escape [103]. Cationic dendrimer NPs with adsorbed antigens demonstrate enhanced delivery of antigens to DCs, and simultaneously activate DCs including the secretion of cytokines such as IL-1β and IL-12 [104]. DCs play a crucial role in the orchestration of the innate and adaptive immune system through antigen uptake, processing, and presentation of epitopes to naive T cells (Figure 3, right). Because most vaccines used in current practice are exogenous to the cells, DCs play a vital role in vaccine-activated cellular immune responses against viral and cancerous diseases. Hence, numerous strategies have been developed for nanovaccine targeting of DCs [70].

DCs express cell-surface mannose receptors which help in antigen internalization through mannosylation, and this enhances the activation of CD4^+^ and CD8^+^ T cell responses [105]. The same strategy has been employed successfully using a dextran-based nanovaccine with lipopolysaccharide (LPS). Nanoformulations showed robust antigen-specific CD4^+^ and CD8^+^ T cell responses, and generated stronger CD8^+^ T cell response than the soluble antigen and LPS mixture [106]. By targeting the langerins (CD207) which are exclusively expressed on Langerhans cells, liposomes conjugated with langerin ligands exhibited effective targeting of Langerhans cells in human skin [107]. In addition to the usual MHC II presentation and CD4^+^ T helper cell activation pathway, DCs can also process antigens and present them via the MHC I pathway leading to activation of CD8^+^ T cell response in a process known as 'cross-presentation' [108,109]. This cross-presentation occurs via the cytosolic pathway. The exogenous antigens are processed in the cytosol by proteasomes [109]. Nanovaccines can modulate intracellular antigen delivery and promote cross-presentation. Many types of NPs including inorganic, polymeric, and lipid NPs were shown to induce effective CD8^+^ T cell expansion by antigen cross-presentation [110,111]. A specially designed polymeric microneedle with encapsulated antigens was able to target Langerhans cells with efficient cross-priming and Th1 immune responses [112]. Cross-presentation was shown to be dependent on the particle–antigen linkage, and disulphide bonding between NPs and antigens results in antigen release into the endosomal compartment leading to subsequent CD8^+^ T cell expansion, whereas non-degradable linkers do not [113].

Other than the cell-mediated immune response, various nanovaccines can elicit humoral responses. B cells, which oversee antibody production, require prolonged and constant activation to generate humoral responses. As mentioned previously, the strategy for loading the antigens onto the NPs may have a profound influence on the resulting humoral responses. For example, calcium phosphate NPs with the antigen covalently attached to the surface exhibit a substantial increase in B cell activation *in vitro* in comparison to the soluble antigen. Similarly, antigen displayed on the surface of multilamellar vesicles provided an enhanced humoral response compared to the encapsulated antigen. However, studies are few and further exploration is warranted. Elevated levels of antigen-specific antibodies can also be achieved by multivalent presentation of antigens, and NP systems can serve as the platform for this purpose. Ueda and colleagues have engineered self-assembling NPs for tailoring the optimal geometry for multivalent presentation of viral glycoproteins [114].

## Strengthening lymph node retention by nanovaccines

The generation of a cell-mediated immune response relies on efficient trafficking or drainage of antigenic components to LNs for further processing and presentation to T and B cells. LNs thus represent a crucial target site for the delivery of vaccines and other immunotherapeutic agents because direct delivery of antigenic components into APCs residing in LNs can induce more potent and robust immune stimulation than can antigen uptake by migrating APCs. LNs also contain a substantial fraction of resident DCs which are phenotypically immature and well equipped for simultaneously internalizing antigens and particles [115]. By targeting LN APCs or DCs instead of those in peripheral sites, immune tolerance as a result of antigen exposure on the DC surface before reaching the LN can be avoided [116]. In addition, DC-targeting ligands are not a prerequisite because the *in situ* concentration of LN-resident DCs is extremely high [117,118]. Therefore, targeting APCs including DCs in LNs that can be readily taken up into lymphatic vessels and retained in draining LNs is a promising strategy.

As mentioned in the previous section, particle size plays an important role in LN targeting and retention. In one study, a synthetic vaccine NP (SVNP) was developed to improve the targeting and retention efficacy of cancer vaccines [119]. The positively charged SVNPs of varying size upon conjugation with a negatively charged tumor antigen showed rapid migration into LNs, leading to secretion of higher levels of proinflammatory cytokines and type I IFN (IFN-α, IFN-β) [119]. In another study, biodegradable NPs of 20, 45, and 100 nm were used as delivery vehicles to DCs in LNs [84]. It was observed that 20 nm poly(ethylene glycol) (PEG)-stabilized poly(propylene sulfide) (PPS) NPs, which can carry hydrophobic drugs and degrade in an oxidative environment, were taken up readily by lymphatic vessels following interstitial administration with 20 nm and 45 nm particles, and showed enhanced retention in LNs [84]. In another instance, large particles (500–2000 nm) were shown to be mostly internalized by DCs from the site of injection, whereas particles of 20–200 nm and virus-like particles (30 nm) were found in LN- resident DCs and macrophages, indicating free drainage and retention of these particles in LNs [120]. It was shown that biodegradable 20 nm PLGA-b-PEG NPs rapidly drained across proximal and distal LNs with a higher retention time than 40 nm particles, whereas the drainage of 100 nm NPs was negligible [121]. In another study where 25 nm and 100 nm Pluronic-stabilized PSS NPs were intradermally injected, there was ten-fold greater interstitial flow into lymphatic capillaries and associated draining LNs for 25 nm particles than for 100 nm particles [65]. Size-dependent LN targeting was also exhibited by 30 nm and 90 nm AuNPs antigen carriers, and 30 nm particles displayed higher LN retention and accumulation than 90 nm particles [122]. In summary, small particle size is required for efficient penetration of lymphatic vessels and prolonged LN retention. NPs with a size in the 20–200 nm range, which coincides with the sizes of viral particles, can exploit interstitial flow for lymphatic delivery, and in this range smaller NPs tend to accumulate more in the LNs.

## Nanomaterial-mediated inflammation and cytokine release

Nanomaterials are known to boost the immune system and have been used to develop vaccines when conjugated with antigens. We review here cases of inflammation reported in the literature that resulted from inflammatory cytokine release following NP administration. The Th1 or Th2 responses elicited thus caused either an efficient immune response or damage to the host tissue.

The use of a lipid-based particle (ISCOMATRIX) as the adjuvant for a chimeric peptide vaccine containing multiple epitopes of T cell lymphotropic virus (HTLV) type I led to enhanced production of mucosal IgA and IgG2a antibody titers as well as increased IFN-γ and IL-10 production [122]. Carbon NPs containing bovine serum albumin exhibited strong stimulation of IgA antibodies in salivary, intestinal, and vaginal mucosa following oral immunization. They were also capable of inducing Th1 and Th2 responses [123]. Kim and coworkers synthesized synthetic vaccine NPs with a combination of OVA and Toll-like receptor 3 (TLR3). These enhanced antigen uptake by APCs and the secretion of inflammatory cytokines including type I interferon, TNF-α, and IL-6 [119]. *Mycobacterium* *tuberculosis (*MtB) lipids attached to chitosan NPs induce both cell-mediated and humoral immunity leading to enhanced secretion of IgG, IgM, and Th1/Th2 cytokines [123]. Amantadine-coated silver NPs triggered HIV-specific cytotoxic T lymphocyte (CTL) production and eightfold stronger TNF-α production *in vivo* [124].

Multiwalled carbon nanotubes and silica NPs can both activate the NOD-like receptor (NLR) family pyrin domain-containing 3 (NLRP3) inflammasome leading to uncontrolled pathological inflammation. Superparamagnetic iron oxide NPs (SPIONs) showed enhanced activation of inflammatory genes in response to LPS [125]. The PLGA-OVA^+^ A20 nanovaccine maintains immune homeostasis by suppressing Th2 inflammation and promoting the regulatory T cell (Treg) response and IL-10 production in lung airway tissue of an allergic asthma murine model [126].

Synergistic stimulation of the production of IL-1β by some NPs and bacteria induces strong pathological inflammation leading to leukocyte influx, swelling, fever, vasodilation, and inflammation-driven tissue damage [127]. Elevated release of proinflammatory cytokines such as IL-6, TNF-α, IL-12 from APCs was observed after the uptake of DNA-inorganic hybrid nanovaccines (hNVs) [128]. The adjuvants used with NP vaccines such as alum, oil in water emulsions (incomplete Freund's adjuvant), and monophosphoryl lipid A (MPLA) are also sometimes associated with inflammation.

Size-dependent immunogenicity of polystyrene particles carrying CpG oligonucleotides was observed in DCs leading to differential expression of IL-6 and IFN-α. CpG-mediated activation of the MAPK and nuclear factor κB (NF-κB) pathways induced the expression of proinflammatory cytokines (e.g., IL-6, IL-12, and TNF-α) [129]. The Th1 immunostimulatory response thus generated suppressed the Th2 immunoregulatory response [130].

Potential cytotoxicity of CTLs was observed in an overtly activated proinflammatory cytokine (IFN-γ, TNF-α) response following albumin/albiCpG nanocomplex inoculation into mice. Encapsulated OVA polyanhydride NPs boosted the formation of antigen-specific CD8^+^ T cell memory after vaccination [131]. Subcutaneous delivery of polyanhydride NPs induced only a mild inflammatory response with no tissue damage [132]. Hyperactivation of the inflammatory response impaired the trafficking, maturation, activation, and memory cell formation of CD8^+^ T cells [133]. More efficient administration of vaccine (e.g., DC-based vaccines, antigen-coated particle formulations) leading to an absence of overt inflammation induced the formation of memory CD8^+^ T cell more effectively following antigen delivery [134].

## Nanovaccines in clinical use and in clinical trials

Only a few nanovaccines have been successfully translated from the laboratory to the clinics. Of these, most only elicit humoral responses, and there is an unmet need for the development of vaccines that can generate strong cellular responses against infectious diseases and cancer. Vaxfectin® is a cationic liposomal nanovaccine which is currently in clinical trials. Vaxfectin® has been used against herpes simplex virus type 2 (HSV-2) and also against influenza virus (H5N1) [135]. Similarly, another FDA-approved nanovaccine, Inflexal®V, has been used as a subunit influenza vaccine in which the hemagglutinin (HA) surface molecules of influenza virus are conjugated to lipid components [136]. Stimulax®, a liposomal therapeutic nanovaccine that is currently under clinical investigation, has been employed as vaccine against cancer [137]. Epaxal is another liposome-based nanovaccine against hepatitis A infection [138].

**Figure 4:**
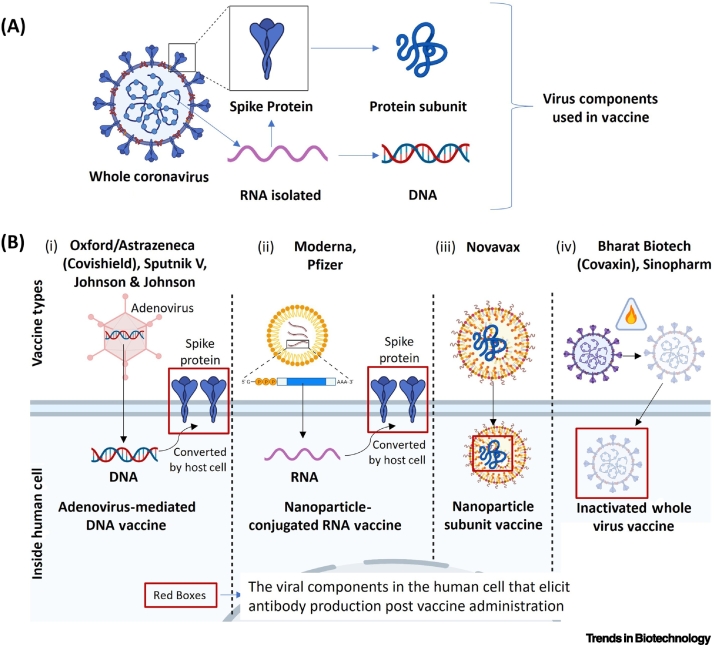
Strategies for the development of nanovaccines against SARS-CoV-2. (A) The spike protein S that is present at the surface of the virus is unique for SARS-CoV-2 and has been used as a vaccine target by different laboratories. Nanovaccines comprise S protein mRNA. although the corresponding DNA sequence can also used. S proteins are often broken down into fragments that can also be used as antigens. (B) (i) The Astrazeneca, Sputnik V, and Johnson & Johnson vaccines use conventional adenovirus-mediated DNA transfer method to express SARS-CoV-2 S protein at the site of inoculation. (ii) The Moderna and Pfizer vaccines introduce S mRNA by means of lipid nanoparticles, leading to local synthesis. (iii) Novavax contains S protein embedded in a nanoparticle system, whereas (iv) Bharat Biotech and Sinopharm used a conventional inactivated whole virus vaccine. Abbreviation: SARS-CoV-2, severe acute respiratory syndrome coronavirus-2.

Significant attention to NPs has been recently drawn during the development of effective vaccines against severe acute respiratory syndrome coronavirus-2 (SARS-CoV-2) (Figure 4). Synchronized innate and adaptive (both humoral and cell-mediated) immune responses are essential for achieving virus clearance from the host. The use of NPs to achieve this goal is generally essential, and a list of SARS-CoV-2 vaccines that take advantage of nanomaterials is provided in Table 2.

**Table 2:** Nanovaccines approved or in clinical trials^a^

Organization	Name of vaccine	Type of antigen	Nanomaterial used	Clinical trial (registration number)	Refs
Moderna and NIAID	mRNA-1273 LNP	mRNA-1273 mRNA	LNP with mRNA encapsulated	Phase I (NCT04283461) Phase II (NCT04405076) Phase III (NCT04470427)	[139,140]
BioNTech and Pfizer	mRNA BNT162b2	mRNA encoding the trimerized RBD of SARS-CoV-2	LNP with mRNA encapsulated	Phase I/II (UTRN U1111-1249-4220) Phase I/II (Germany) NCT04537949 EudraCT Number (Germany) (2020–001038-36) Phase II/III (USA) (NCT04368728) Phase I/II (ChiCTR2000034825) Phase I (Japan) NCT04588480	[141,142]
Novavax	NVX-CoV2373	Full-length SARS-CoV-2 S glycoprotein	Recombinant glycoprotein NP saponin-based Matrix-M1 adjuvant	Phase I (NCT04368988) Phase II (NCT04533399) Phase III (UK) (2020–004123-16) Phase III (USA/Mexico) NCT04611802	[143]
Imperial College, London Acuitas Therapeutics, Vancouver	LNP-nCoV saRNA ARCT-021	saRNA and pre-fusion stabilized SARS-CoV-2 S protein	LNP with saRNA encapsulated	ISRCTN1707269, NCT04480957	
Suzhou Abogen Biosciences Walvax Biotechnology and People's Liberation Army	ARCoV	mRNA encoding RBD of SARS-CoV-2 S glycoprotein	LNP with mRNA encapsulated	Phase I (ChiCTR2000034112)	
Novavax		SARS-CoV S protein and influenza M1 protein	SARS-CoV VLP nanovaccine	Preclinical	
Imophoron and Bristol University		Multiepitope display	VLP ADDomer™	Preclinical	http://www.bristol.ac.uk/news/2020/april/covid-19-vaccine-platform.html
Fundan University, Shanghai JiaoTong University, and RNACure Biopharma		mRNA cocktail	LNP with VLP encapsulated	Preclinical	[144]
Crucell	Inflexal®V	Influenza	Virosome with influenza virus surface antigens (hemagglutinin and neuraminidase)	Phase III completed NCT01631110	EMA
Crucell	Epaxal®	Hepatitis A	Virosome with inactivated virus particles	Phase III completed NCT01307436	EMA
Merck	Gardasil®9	HPV	Capsomere (major capsid protein L1)	Completed NCT00090220	FDA EMA
GSK	Cervarix®	HPV	Capsomere (major capsid protein L1)	Phase II NCT00316693 Phase III NCT03728881	FDA EMA
Dendreon Pharmaceuticals	Provenge (Sipuleucel-T)	Prostate cancer	Each dose of contains a minimum of 50 million autologous CD54^+^ cells activated with PAP-GM-CSF	Phase III completed NCT00065442	FDA
Novavax	NanoFlu^TM^	Influenza	Recombinant HA protein on Tween 80 NP with Matrix-M adjuvant	Phase III NCT04120194	Active
Novavax	ResVax^TM^	RSV (protection of infants via maternal immunization)	Recombinant near full-length RSV F protein on Tween 80 NP with/without alum adjuvant	Phase III NCT02624947 2016-002302-39	Completed (2020)
Novavax	ResVax^TM^ (coadministration with influenza vaccine)	RSV and influenza (≥60 years of age)	Recombinant near full-length RSV F protein on Tween 80 NP with/without alum adjuvant coadministered with licensed influenza vaccine	Phase I NCT01709019	Completed (2014)
Novavax	EBOV GP Vaccine	Ebola	2014 Guinea Ebola virus recombinant glycoprotein on Tween 80 NP with/without Matrix-M adjuvant	Phase I NCT02370589	Completed (2016)
Sensei Biotherapeutics	PAN-301-1	Prostate cancer	Modified bacteriophage (viral NP) expressing 200–300 copies of part of the human aspartyl (asparaginyl) β-hydroxylase molecule on the viral head	Phase I NCT03120832	Completed (2019)
DAIDS/NIAID/NIH	MPER-656	HIV	HIV-1 gp41 membrane proximal external region (MPER) with liposomes	Phase I NCT03934541	Active
BioNTech	W_ova1	Ovarian cancer	Liposome-formulated mRNAs. Three ovarian cancer tumor-associated antigens in combination with (neo-)adjuvant chemotherapy	Phase I NCT04163094	Active
ImmunoVaccine Technologies	DPX-0907	Ovarian, breast, and prostate cancer	Liposomes with seven tumor-specific HLA-A2-restricted peptides, a universal T helper peptide, and a polynucleotide adjuvant in Montanide ISA51 VG	Phase I NCT01095848	Completed (2015)
Merck	Tecemotide	Multiple myeloma	Liposomes with tecemotide lipopeptide and 3-*O*-deacyl-4′-monophosphoryl lipid adjuvant	Phase II NCT01094548	Completed (2016)
		NSCLC (discontinued)		Phase I/II NCT00960115 Phase III NCT01015443 NCT02049151 NCT00409188	Completed (2015) Terminated for NSCLC indication
		NSCLC (bevacizumab)		Phase II NCT00828009	Active
		Breast cancer		Phase III NCT00925548	Terminated (clinical hold)
		Colon/rectum carcinoma		Phase II NCT01462513	Completed (2018)
		Prostate cancer		Phase II NCT01496131	Completed (2018)
		Rectal cancer		Phase II NCT01507103	Completed (2017)
Cascadian Therapeutics	ONT-10	Solid tumor	Liposomal MUC1 cancer vaccine	Phase I NCT01556789 NCT01978964	Completed (2018) (2018)
XEME Biopharma	Oncoquest^TM^	Follicular lymphoma Chronic lymphocytic leukemia	Liposomes containing autologous tumor-derived antigen and IL-2	Phase I NCT01976520 Phase II NCT02194751	Active Active
Lipotek Pty	Lipovaxin-MM	Metastatic melanoma	Multicomponent liposomes containing tumor antigens (gp100, tyrosinase, and melanA/MART-1) with DC-targeting moiety DMS-5000	Phase I NCT01052142	Completed (2012)
NasVax	VaxiSome^TM^-Influenza	Influenza	VaxiSome^TM^ (ceramide carbamoyl-spermine/cholesterol) liposomal adjuvant/delivery system combined with commercial influenza vaccine	Phase II NCT00915187	Completed (2011)

a Abbreviations: ARE, *Asparagus racemosus* extract; CsAuNPs, chitosan-functionalized AuNPs; F, RSV fusion protein; HA, influenza virus hemagglutinin; HPV, human papillomavirus; LNP, lipid nanoparticle; melanA/MART-1, melanoma antigen recognized by T cells; MUC1, mucin 1; NSCLC, non-small cell lung cancer; PAP-GM-CSF, pulmonary alveolar proteinosis granulocyte macrophage colony-stimulating factor; RBD, receptor-binding domain; RSV, respiratory syncytial virus; S, SARS-CoV-19 spike protein; saRNA, self-amplifying mRNA; VLP, vaccine-like particle.

Boston-based Moderna in conjunction with the National Institute of Allergy and Infectious Diseases (NIAID) developed a mRNA-based NP vaccine against SARS-CoV-2 [139]. The mRNA contains the coding sequence for SARS-CoV-2 spike (S) protein and is encapsulated within lipid NPs that induce efficient uptake by immune cells and the activation of T and B lymphocytes [139]. An adaptive immune response is thus generated against the S protein [139,140]. Pfizer and BioNTech jointly developed the BNT162 (b1, b2) vaccine against SARS-CoV-2. BNT162b1 is mRNA-based vaccine that encodes a trimer of the viral receptor-binding domain (RBD) [141]. BNT162b2 is another mRNA vaccine which codes for full-length membrane-anchored S protein [142]. Both mRNAs are encapsulated in lipid NPs for efficient delivery into target cells. The mRNA sequences are partially modified to enhance RNA stability and protect the RNA conformation to improve immunogenicity [141,142]. The Moderna and BNT vaccines were the among the first approved vaccines against SARS-CoV-2. Maryland-based Novavax expressed full-length SARS-CoV-2 S glycoprotein in a baculovirus/Sf9 system. The saponin-based Matrix-M1 adjuvant is used during administration, which overcomes the problem of not inducing a cell-mediated immune response observed with other protein subunit vaccines [143]. The Novavax vaccine is currently under review for emergency use authorization (EUA).

In addition to SARS-CoV-2, the use of nanovaccines is widespread in multiple other diseases as well. Many of them have been approved by FDA and/or European Medicines Agency (EMA) and others are currently in clinical trials. A list of such vaccines is provided in Table 2.

## Concluding remarks and future perspectives

Despite advances in the development of traditional vaccines, improvements are needed because of the weak immunogenicity of conventional vaccines, intrinsic instability *in vivo*, toxicity, and the need for multiple booster immunizations. Nanovaccines, which are the focus of this review, provide distinct advantages over conventional vaccines because of their size proximity to pathogens, controllable physicochemical and biophysical attributes, enhanced protection of the antigen from degradation, biopersistence, improved transport through the lymphatics and into LNs, and codelivery of immunomodulatory molecules to boost immune recognition (Box 1, Box 2).

Box 2 **The future of nanovaccines**

Personalized nanovaccines

Different immunization efficiencies have been reported in different groups (young/adults, diabetic/non-diabetic, male/female, etc.) during the development of different coronavirus disease 2019 (COVID-19) vaccines. Nanotools can offer a range of advanced strategies to potentially develop a seasonal vaccine where one infection may possibly facilitate other infections (e.g., influenza infection can facilitate bacterial superinfection and pneumonia; coinfection with influenza A virus can enhance SARS-CoV-2 infectivity). It remains to be determined whether nanovaccines with multiple epitopes and/or adjuvants can be developed to generate broad-spectrum immunity. Nanotools offer the best possible non-viral strategy to encapsulate and deliver nucleic acids (including plasmids and mRNA), although thermostability remains an unresolved issue. It is well established that the immune system is differently configured in different individuals, and a 'one-size-fits-all' approach is not an optimal solution – nanovaccines hold potential in the development of a new frontier of personalized vaccines that will ensure wider and longer-term protection (Figure I).

**Figure I:**
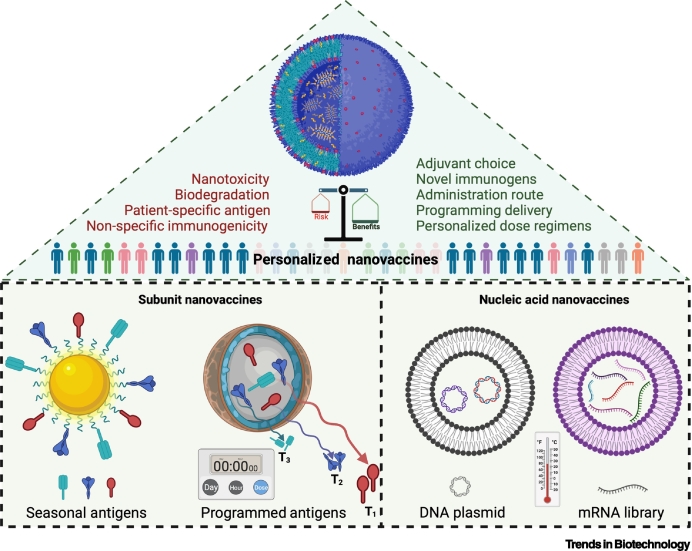
Future nanovaccines.

Alt-text: Box 2

Recent advances in nanoengineering have played a pivotal role in developing the highly anticipated liposome-based mRNA vaccine against SARS-CoV-2. Nevertheless, there are unanswered challenges in the path of successful translation of various nanovaccines. The nanoscale size range of the antigen vehicle is a crucial criterion which determines the spatial location of the antigen. The optimum size is not generalizable and depends on several factors such as the chemical composition of the nanovaccine and opsonization by complement and complement receptors. Understanding how nanovaccines elicit clonal bursts and somatic hypermutation needs to be addressed for the design of improved nanovaccines against highly variable viruses such as SARS-CoV-2 and influenza, where the success of immunization depends on eliciting extensive somatic hypermutation in antibody-secreting B cells.

Finally, the promise of nanovaccines does not end with the simple induction of humoral or cell-mediated immunity, and nanovaccines represent a new frontier in the development personalized vaccines (Box 2). However, many issues remain unresolved (see Outstanding questions) and a risk–benefit analysis is required. Once preclinical studies are validated in animal models, clinical translation of nanovaccines will require stringent safety testing to address different types of risks and scenarios (Box 2). In addition, setting up an analytical pipeline for the development of nanovaccines of different compositions will require further systematic investigations.

Outstanding questions Can nanoscale materials be used to facilitate vaccine development?

How do nanoscale properties such as size, shape, geometry, and surface functionalization contribute toward an effective immune response?

How do nanovaccines complement the vaccine development process in the current pandemic scenario?

Is it possible to acquire and track indicators of the long-term impact of nanovaccines over the lifetime of an individual?

Alt-text: Outstanding questions
